# Enhancement of Bentonite Materials with Cement for Gamma-Ray Shielding Capability

**DOI:** 10.3390/ma14164697

**Published:** 2021-08-20

**Authors:** Ahmed M. El-Khatib, Mohamed Elsafi, Mohamed N. Almutiri, R. M. M. Mahmoud, Jamila S. Alzahrani, M. I. Sayyed, Mahmoud I. Abbas

**Affiliations:** 1 Physics Department, Faculty of Science, Alexandria University, Alexandria 21511, Egypt; elkhatib60@yahoo.com (A.M.E.-K.); mgggm858@gmail.com (M.N.A.); mabbas@physicist.net (M.I.A.); 2 Physics Department, College of Science and Arts in Almithnab, Al-Qassim University, Al Mulaida, Buraydah 52571, Saudi Arabia; 3 Radiation Protection Department, Nuclear and Radiation Safety Research Center, Atomic Energy Authority, Cairo 11762, Egypt; randam76@yahoo.com; 4 Physcis Department, College of Science, Princess Nourah Bint Abdulrahman University, Riyadh 11671, Saudi Arabia; jsalzahrani@pnu.edu.sa; 5 Department of Physics, Faculty of Science, Isra University, Amman 11622, Jordan; dr.mabualssayed@gmail.com; 6 Department of Nuclear Medicine Research, Institute for Research and Medical Consultations (IRMC), Imam Abdulrahman bin Faisal University (IAU), P.O. Box 1982, Dammam 31441, Saudi Arabia

**Keywords:** bentonite–cement mixed materials, MFP, XCom software, mass attenuation coefficient

## Abstract

The gamma-ray shielding ability of various Bentonite–Cement mixed materials from northeast Egypt have been examined by determining their theoretical and experimental mass attenuation coefficients, μ_m_ (cm^2^g^−1^), at photon energies of 59.6, 121.78, 344.28, 661.66, 964.13, 1173.23, 1332.5 and 1408.01 keV emitted from ^241^Am, ^137^Cs, ^152^Eu and ^60^Co point sources. The μ_m_ was theoretically calculated using the chemical compositions obtained by Energy Dispersive X-ray Analysis (EDX), while a NaI (Tl) scintillation detector was used to experimentally determine the μ_m_ (cm^2^g^−1^) of the mixed samples. The theoretical values are in acceptable agreement with the experimental calculations of the XCom software. The linear attenuation coefficient (μ), mean free path (MFP), half-value layer (HVL) and the exposure buildup factor (EBF) were also calculated by knowing the μ_m_ values of the examined samples. The gamma-radiation shielding ability of the selected Bentonite–Cement mixed samples have been studied against other puplished shielding materials. Knowledge of various factors such as thermo-chemical stability, availability and water holding capacity of the bentonite–cement mixed samples can be analyzed to determine the effectiveness of the materials to shield gamma rays.

## 1. Introduction

Egypt is looking at nuclear technology to generate and improve the electric energy provided by the nuclear reactors at El Dabaa [1]. Besides increasing the use of radioisotopes and photon-emitting tools in nuclear and industrial fields, it is necessary to examine the shielding capacity of gamma radiation shields for their use in construction, such as a bentonite–cement mixture. Bentonite material is such a clay used for building and construction purposes in different countries. The products of clay such as tiles, baked bricks, and crockery are more durable and cheaper building materials than cement, especially in tropical conditions [2].

It is indisputable that ionizing radiation can be harmful if humans are exposed to it for a long period of time. Therefore, the main function of radiation shields is to reduce the intensity of the emitted photons to an acceptable level, considering the risk which may be caused by exposure to ionizing photons [3,4,5]. It was found that bentonite has very good thermal properties: thermo-chemical stability, melting point is high, high thermal shock resistance, at high temperature has a good mechanical strength, low thermal shrinkage and high corrosion resistance [6]. Bentonite is also a highly available composite material, thus proving to be cost-effective, environmentally friendly and non-toxic. These characristics make clay materials composite suitable for taking acount in shielding applications.

Since γ-rays do not have charges, in addition to their penetration power, a safe and protective shield must be made for this radiation. The photon interaction with matter depends on the energy of the photons upon impact, divided into three regions of energy: at low energy, the photoelectric effect is dominant interaction, at medium energy, the dominant interaction is Compton scattering, and pair production is dominant with high energy photons. The total probability of this interaction with matter through path-length is the linear attenuation parameter μ (cm^−1^). The absorber sample also depends on its density ρ (g·cm^−3^). Therefore, the more fundamental attenuation value is called the mass attenuation coefficient μ/ρ (cm^2^·g^-1^) because it does not effected by the physical state and density of the studied sample.

There are other coefficients used to check the shielding ability of the studied samples, such as the mean free path (MFP), half-value layer (HVL) and exposure buildup factor (EBF). The MFP measures the average distance of radiation traveled through the absorber without any interaction, while the HVL measures the thickness layer needed to reduce the radiation intensity to half of its initial intensity and the EBF measures the probability of scattering radiation from an absorber, and its values must be greater than or equal 1 (EBF ≥ 1, EBF = 1 IF total absorption occurred). These parameters have been calculated for many different materials used for shielding applications [7,8,9,10,11,12,13,14,15,16].

The current study aims to study the γ-ray shielding capability for Egyptian bentonite clays enhanced by some composites. The mass attenuation coefficient was theoretically and experimentally determined at different energies, and from these results, the MFP and HVL were calculated. The G-P fitting parameters are used to evaluate the EBF for the present samples. There is a clear difference between the present study and the research presented by S. Asal et al., 2021 [17], which studied the natural bentonite clays without any additives, while in this work, the bentonite has been improved by cement to increase its density and hardness at the same time. The main purpose of this research is to replace bentonite instead of sand that is mixed with cement to form a mortar layer, and this is a very useful and important application, especially in X-ray chambers to absorb low energies. On the other hand, it is manufactured building blocks for places that work with radiation.

## 2. Materials and Methods

### 2.1. Shielding Parameters

To evaluate the interaction between gamma radiation and incident matter, the linear attenuation coefficient (μ) is a key parameter and can be calculated by Beer–Lambert’s Law [18] as follows:

(1) $$\mu =\frac{1}{x}\ln\left(\frac{I}{I_{0}}\right)$$

where I_0_ is the intensity of incident γ-ray photon while I is transmitted γ-ray photons through a target of absorber thickness x. I and I_0_ were calculated by determining the peak count rate in the presence and absence of the bentonite sample, respectively.

The mass attenuation coefficient (μ/ρ) was calculated to check the ability of the studied materials as shielding to rays without depending on the density of the material, by dividing the experimental calculated (μ) for a given material by its density (ρ). The (μ/ρ) can also be calculated theoretically using Equation (2) [19]:

(2) $$\frac{\mu }{\rho }=\underset{i}{\sum }w_{i}{\left(\frac{\mu }{\rho }\right)}_{i}$$

where (μ/ρ)_i_ and (w_i_) are the mass attenuation and the weight-fraction of the ith constituent element in the sample, respectively. 

The half-value layer (HVL) is an important parameter when making a siutable radiation protecting material. This factor is the absorption thickness required to decrease the incident radiation to 50% of its initial value and is evaluated using Equation (3) [20]:

(3) $$\mathrm{HVL}=\frac{\ln2}{\mu }$$

When the photons pass through the sample, they travel a certain distance; the middle distance that a radiation travels between two consecutive interactions is known as the MFP and is described by Equation (4) [21]:

(4) $$\mathrm{MFP}=\frac{1}{\mu }$$

When designing and selecting the shielding material, the EBF and EABF should be taken into account to correct the attenuation calculations due to the buildup of secondary photons generated by Compton scattering [22]. The minimum value of the buildup factor is 1 (BF ≥ 1); in this case, the absorption ratio of the buildup photons is 100%, and the greater the buildup factor more than one, the higher the scattering ratio of the buildup photons. Both exposure and energy absorption buildup factor can be estimated by phy-x software depending on the chemical composition of sample and its density [23].

### 2.2. Experiment

Bentonite clay was collected from (Suez-city) northeastern Egypt and can easily be obtained because it is abundantly available in Egypt. It was also cut into pieces, crushed into a suitable shape and then dried. A quantity of ordinary cement was also collected. Each type was grounded for a boundary and sifted with a 50 μm mesh sieve. Bentonite–cement mixed materials were studied with different percentages of bentonite. The first sample contains 70% bentonite and 30% cement. A small oven was utilized to bake the studied samples at temperatures of 500 °C. The first sample of a 70% bentonite−30% cement mixture labeled as Form B-C1 for non-baked and B-C2 for that which was baked at 500 °C, respectively. Meanwhile, the second sample of a 50% bentonite−50% cement mixture was labeled as B-C3 for non-baked and B-C4 for that which was baked at 500 °C, respectively.

To identify the elemental compositions of the present samples, EDX or Energy Dispersive X-ray Analysis (JSM-5300, JEOL Ltd., Tokyo, Japan) was used. EDX systems are attachments to Electron Microscopy instruments (Scanning Electron Microscopy (SEM). The compositions have been tabulated in Table 1. By knowing these compositions, the mass attenuation coefficient can be calculated using WinXCom program.

**Figure 1:**
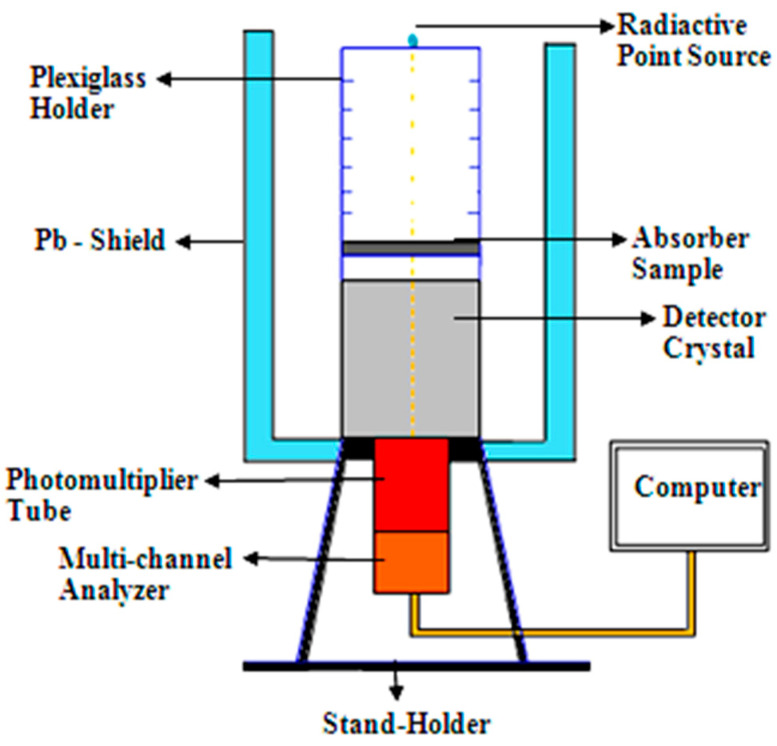
Illustration of the experimental setup.

For the experimental measurements, different radioactive point sources with different energies are used and their characteristics are listed in Table 2. A NaI (Tl) scintillation detector was used to detect the radiation with and without the absorber sample. The spectrum is obtained using Ganei 2000 software, and the net count rate was determined at certain photon energies. The detector was shielded through the measurements and the absorber sample was placed at the top of the detector. The point source was placed in a suitable axial position using a plexiglass holder to avoid the coincidence summing effect for multi-line emitted source and reduce the dead time of the detector as possible. The experimental setup was illustrated in Figure 1.

## 3. Results and Discussion

The chemical compositions of the prepared samples were analyzed using EDX, and the results are tabulated in Table 1. Using the previously mentioned Equation (1), the mass attenuation coefficient can be calculated for any absorber sample. The WinXcom software was also utilized by inputting the chemical composition of the samples to calculate their mass attenuation coefficient. The uncertainty of the mass attenuation coefficient is estimated by the following equation:

(5) $$\sigma_{\mu_{m}}=\mu_{m}\cdot \sqrt{{\left(\frac{\partial \mu_{m}}{\partial N}\right)}^{2}\cdot \sigma_{N}^{2}+{\left(\frac{\partial \mu_{m}}{\partial d}\right)}^{2}\cdot \sigma_{d}^{2}}$$

where $\sigma_{N}$ and $\sigma_{d}$ are the uncertainty in count rate and mass thickness (g/cm^2^), respectively. In addition to the experimental method, the mass attenuation coefficient was also theoretically calculated and was found that to be in good agreement with the experimental values, as shown in Table 3.

**Figure 2:**
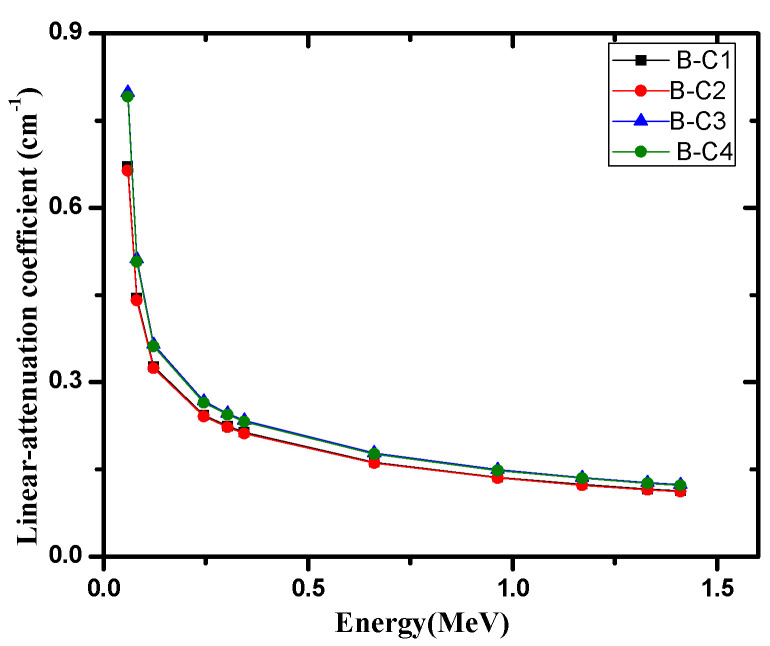
The linear attenuation coefficient of the four investigated samples at different energies.

**Figure 3:**
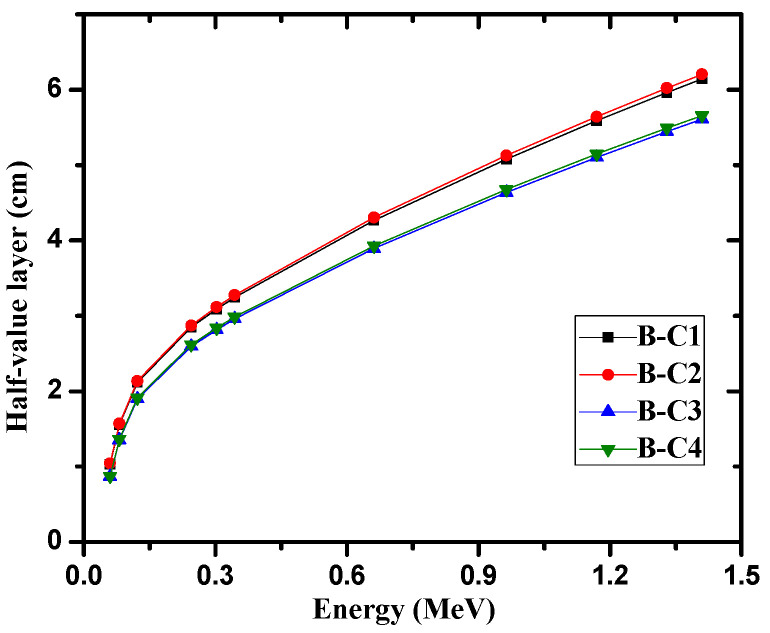
The Half-value layer of the four investigated samples at different energies.

**Figure 4:**
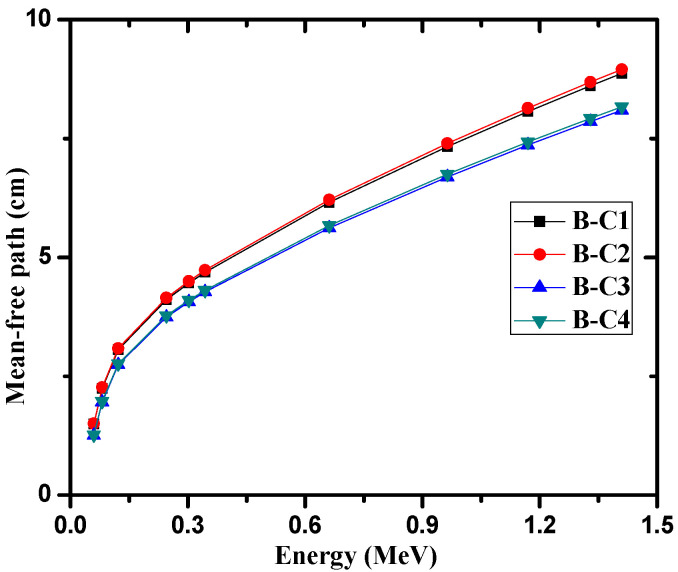
The mean-free path of the four investigated samples at different energies.

From these results, it is clear that the effect of raising the temperature had practically no effect on the mass attenuation coefficients of the present samples. The linear attenuation coefficient (see Figure 2) is a valuable parameter that can be used to calculate other important shielding parameters can be calculated. The HVL and MFP were calculated and analyzed for the investigated samples in Figure 3 and Figure 4, respectively. The increase of cement in the samples proved to improve the shielding ability of the samples, as B-C3 attenuated more radiation than the B-C1 sample, and the thickness needed to reduce the intensity of 1.41 MeV gamma rays to one-half of its initial intensity is about 5.6 cm for the B-C3 sample, while about 6.1 cm are required for B-C1 at the same energy. The average distance gamma rays traveled between two successive interactions at 1.41 MeV for the B-C1 sample is about 8.8 cm, while it is about 8 cm for the B-C3 sample. The present samples were compared with the similar previously studied shielded materials such nature bentonite samples [24], bentonite with steel slag [25], ordinary concrete and steel scrap [26] and ball and kaolin clays [27] at two different high energies, 1.173 and 1.332 MeV, and the results are listed in Table 4.

**Figure 5:**
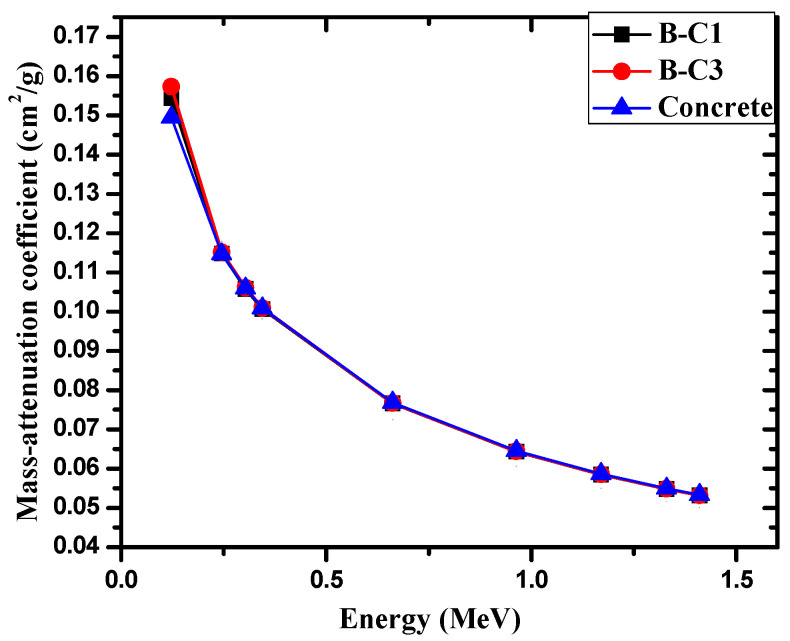
The compared mass attenuation coefficient of the studied shielded samples with iron at different energies.

The mass attenuation coefficient for the compared shielding materials is almost the same, as shown in Table 4, because this parameter is not affected by the density of the absorber material. The results demonstrated that the current samples have the same shielding ability as ordinary concrete and the same thickness needed to reduce the initial intensity of the incoming radiation in half and to one-tenth of its original value (HVL and TVL, respectively). The μ/ρ of the studied shielded samples was also graphed against concrete in Figure 5.

**Figure 6:**
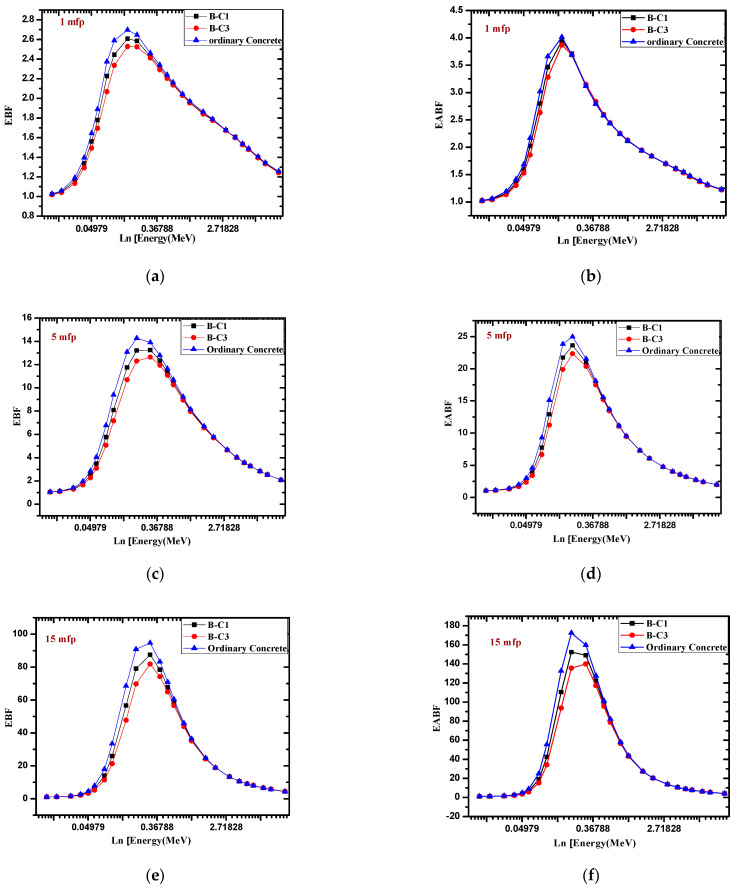
The EBF and EABF variation against different energy in log scale of the present samples compared with concrete at 1 mfp, 5 mfp and 15 mfp. (**a**): EPF at 1 mfp; (**b**): EAPF at 1mfp; (**c**): EPF at 5 mfp; (**d**)**:** EAPF at 5 mfp; (**e**): EPF at 15 mfp; (**f**): EAPF at 15 mfp.

**Figure 7:**
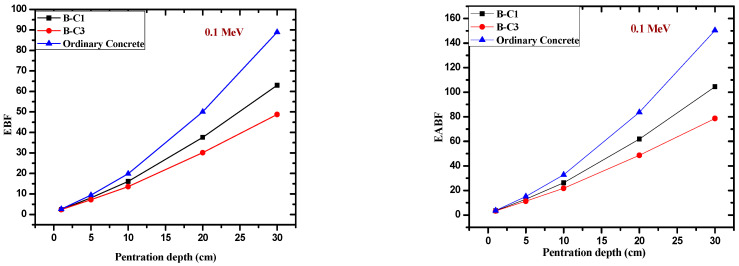
The variation of the EBF and EABF against penetration depth of the present samples compared with concrete at 0.1 MeV.

The energy absorption and the exposure buildup factor of the present samples were calculated by using the G-P fitting parameters programmed by phy-x software, which depend on the Compton and total attenuation of samples as well as the chemical composition of samples. The EBF and EABF of present samples were compared with concrete [28] at different mean free paths (mfps) with different energies, ranging from low to high energies, in Figure 6. The variation of EBF and EABF with energy in the selected samples indicated that the present samples have a lower EBF and EABF than ordinary concrete at intermediate energies, while at low and high energy, the EBF and EABF have the same approximate values. In addition, the variation of EBF and EABF with a penetration depth of the present samples was compared with concrete at 0.1 MeV, as shown in Figure 7. This figure indicates that the EBF and EABF of the present samples at different penetration depths are less than concrete’s buildup factors with increasing mfp, while at low mfp, the effect of EBF and EABF of the present samples are nearly similar with concrete.

## 4. Conclusions

The gamma-ray shielding ability of modified bentonite clay samples with cement ratios was studied experimentally and compared theoretically using WinXcom software. The experimentally determined mass attenuation coefficients are consistent with the values evaluated by WinXcom. The obtained mass attenuation coefficients, linear attenuation coefficients, HVL and TVL were compared with the values of other studied materials and found to be better and comparable with the selected materials. EBF and EABF are important factors for radiation protection applications, and the present work indicated that EBF and EABF for the studied samples are lower than EBF and EABF for concrete as well as more desirable. Due to factors such as cost, availability, thermochemical stability and energy ranging from 0.1 to 3 MV, it has been concluded that cement-reinforced bentonite materials can be used for gamma-radiation protection in both nuclear and medical applications.

## Figures and Tables

**Table 1:** The chemical composition of present samples.

Component	SampleB-C1 Density (g/cm^3^) = 2.12 Mass (%)	SampleB-C2 Density (g/cm^3^) = 2.10 Mass (%)	SampleB-C3 Density (g/cm^3^) = 2.32 Mass (%)	SampleB-C1 Density (g/cm^3^) = 2.30 Mass (%)
SiO_2_	49.490	49.543	41.430	41.494
Al_2_O_3_	14.273	14.261	11.795	11.785
CaO	25.370	25.375	36.590	36.598
Fe_2_O_3_	3.780	3.773	3.660	3.652
SO_3_	0.642	0.622	1.070	1.033
Na_2_O	2.325	2.323	1.675	1.674
MgO	2.321	2.317	2.275	2.270
TiO	0.087	0.085	0.145	0.142
K_2_O	1.712	1.701	1.360	1.351
Total	100.00	100.00	100.00	100.00

**Table 2:** The radioactive point sources and their characteristics which are used in the present work.

Reference Date	Energy keV	Activity kBq	Uncertainty kBq	Emission Probability %	PTB Nuclide
1 June 2009	59.52	259.0	±2.6	35.9	^241^Am
	80.99	275.3	±2.8	34.1	^133^Ba
	121.78	290.0	±4.0	28.4	^152^Eu
	244.69			7.49	
	344.28			26.6	
	964.13			14.0	
	1408.01			20.87	
	661.66	385.0	±4.0	85.21	^137^Cs
	1173.23	212.1	±1.5	99.9	^60^Co
	1332.50			99.982	

**Table 3:** The theoretical and experimental mass attenuation coefficient.

Energy (keV)	Experimental μ_m_ (cm^2^/g)				Theoretical μ_m_ (cm^2^/g)			
	B-C1	B-C2	B-C3	B-C4	B-C1	B-C2	B-C3	B-C4
59.53	0.3130 ± 0.0021	0.3129 ± 0.0019	0.3398 ± 0.0020	0.3455 ± 0.0028	0.3162	0.3162	0.3441	0.3440
80.99	0.2068 ± 0.0020	0.2059 ± 0.0025	0.2162 ± 0.0025	0.2218 ± 0.0021	0.2100	0.2099	0.2206	0.2205
121.78	0.1528 ± 0.0022	0.1511 ± 0.0018	0.1554 ± 0.0021	0.1582 ± 0.0022	0.1543	0.1543	0.1573	0.1573
244.69	0.1138 ± 0.0025	0.1134 ± 0.0032	0.1145 ± 0.0027	0.1153 ± 0.0027	0.1148	0.1148	0.1151	0.1151
302.85	0.1045 ± 0.0030	0.1053 ± 0.0019	0.1060 ± 0.0028	0.1061 ± 0.0034	0.1058	0.1058	0.1061	0.1061
344.28	0.1003 ± 0.0031	0.1017 ± 0.0024	0.1007 ± 0.0028	0.1010 ± 0.0024	0.1007	0.1007	0.1009	0.1009
661.66	0.0763 ± 0.0028	0.0763 ± 0.0028	0.0767 ± 0.0031	0.0774 ± 0.0027	0.0766	0.0766	0.0767	0.0767
964.13	0.0639 ± 0.0025	0.0643 ± 0.0027	0.0644 ± 0.0035	0.0637 ± 0.0029	0.0644	0.0644	0.0644	0.0644
1173.23	0.0580 ± 0.0025	0.0580 ± 0.0029	0.0580 ± 0.0032	0.0584 ± 0.0032	0.0585	0.0585	0.0585	0.0585
1332.5	0.0547 ± 0.0027	0.0542 ± 0.0031	0.0547 ± 0.0031	0.0544 ± 0.0020	0.0548	0.0548	0.0549	0.0549
1408.01	0.0527 ± 0.0030	0.0529 ± 0.0024	0.0527 ± 0.0020	0.0526 ± 0.0028	0.0532	0.0532	0.0532	0.0532

**Table 4:** Comparison of the shielding ability of the present samples against different studied materials.

Absorber Sample		Density (g/cm^3^)	1.173 MeV				1.332 MeV			
			μ_m_ (cm^2^/g)	μ (cm^−1^)	HVL (cm)	TVL (cm)	μ_m_ (cm^2^/g)	μ (cm^−1^)	HVL (cm)	TVL (cm)
**[24]**	Natural bentonite	0.85	0.061	0.052	13.330	44.280	0.049	0.049	14.146	46.991
**[25]**	Bentonite with steel slag	1.98	0.056	0.110	6.300	20.936	0.050	0.100	6.930	23.026
**[26]**	Ordinary concrete	2.30	0.059	0.137	5.072	16.835	0.056	0.128	5.419	17.986
	Steel scrap	4.00	0.057	0.228	3.034	10.070	0.054	0.214	3.238	10.748
**[27]**	Ball clay	1.99	0.060	0.120	5.794	19.231	0.057	0.112	6.164	20.456
	Kaolin clay	1.99	0.060	0.120	5.794	19.231	0.057	0.112	6.164	20.456
**Current work**	B-C3	2.32	0.058	0.136	5.111	16.964	0.055	0.127	5.455	18.104
	B-C1	2.12	0.058	0.124	5.590	18.552	0.055	0.116	5.965	19.798

## Data Availability

The data presented in this study are available on request from the corresponding author.

## References

[1] https://www.neimagazine.com/news/newsconstruction-ofegypts-first-nuclear-plant-to-begin-in-(2021)-8100216.

[2] Nnuka E., Enejor C. (2001). Characterisation of Nahuta clay for industrial and commercial applications. Niger. J. Eng. Mater..

[3] Turner J.E. (2007). Atoms, Radiation and Radiation Protection.

[4] Knoll G.F. (2000). Radiation Detection and Measurement.

[5] Hall E.J. (2000). Radiobiology for the Radiologist.

[6] Alkaya D., Esener A.B. (2011). Usability of sand-bentonite-cement mixture in the construction of impermeable layer. Sci. Res. Essays.

[7] Harjinder S.M. (2016). Experimental investigation of clay fly-ash bricks for gammaray shielding. Nucl. Eng. Technol..

[8] Oto B., Gur A. (2013). Gamma-ray shielding of concretes including magnetite in different rate. Int. J. Phys. Sci..

[9] Shamsan S.O., Sayyed M.I., Gaikwad D.K., Pawar P.P. (2018). Attenuation coefficients and exposure buildup factor of some rocks for gamma ray shielding applications. Radiat. Phys. Chem..

[10] Sayyed M., Issa S.A., Büyükyıldız M., Dong M. (2018). Determination of nuclear radiation shielding properties of some tellurite glasses using MCNP5 code. Radiat. Phys. Chem..

[11] Taqi A.H., Khalil H.J. (2017). Experimental and theoretical investigation of gamma attenuation of building materials. J. Nucl. Part. Phys..

[12] Sayyed M.I., Çelikbilek Ersundu M., Ersundu A.E., Lakshminarayana G., Kostka P. (2018). Investigation of radiation shielding properties for MeOPbCl_2_-TeO_2_ (MeO ¼ Bi_2_O_3_, MoO_3_, Sb2O_3_, WO_3_, ZnO) glasses. Radiat. Phys. Chem..

[13] Tekin H.O., Sayyed M., Manici T., Altunsoy E.E. (2018). Photon shielding characterizations of bismuth modified borate -silicate-tellurite glasses using MCNPX Monte Carlo code. Mater. Chem. Phys..

[14] Sayyed M., Lakshminarayana G. (2018). Structural, thermal, optical features and shielding parameters investigations of optical glasses for gamma radiation shielding and defense applications. J. Noncryst. Solids.

[15] Dong M., Sayyed M., Lakshminarayana G., Çelikbilek Ersundu M., Ersundu A.E., Nayar P., Mahdi M.A. (2017). Investigation of gamma radiation shielding properties of lithium zinc bismuth borate glasses using XCOM program and MCNP5 code. J. Noncryst. Solids.

[16] Kurudirek M., Chutithanapanon N., Laopaiboon R., Yenchai C., Bootjomchai C. (2017). Effect of Bi2O3 on gamma ray shielding and structural properties of borosilicate glasses recycled from high pressure sodium lamp glass. J. Alloy. Compd..

[17] Asal S., Erenturk S.A., Haciyakupoglu S. (2021). Bentonite based ceramic materials from a perspective of gamma-ray shielding: Preparation, characterization and performance evaluation. Nucl. Eng. Technol..

[18] Alharshan G., Aloraini D., Elzaher M., Badawi M., Alabsy M., Abbas M., El-Khatib A. (2020). A comparative study between nano-cadmium oxide and lead oxide reinforced in high density polyethylene as gamma rays shielding composites. Nucl. Technol. Radiat. Prot..

[19] Mahmoud K., Sayyed M., Tashlykov O. (2019). Gamma ray shielding characteristics and exposure buildup factor for some natural rocks using MCNP-5 code. Nucl. Eng. Technol..

[20] Kiani M.A., Ahmadi S.J., Outokesh M., Adeli R., Kiani H. (2019). Study on physico-mechanical and gamma-ray shielding characteristics of new ternary nanocomposites. Appl. Radiat. Isot..

[21] Rammah Y., Ali A., El-Mallawany R., El-Agawany F. (2020). Fabrication, physical, optical characteristics and gamma-ray competence of novel bismo-borate glasses doped with Yb2O3 rare earth. Phys. B Condens. Matter.

[22] Kurudirek M. (2014). Photon buildup factors in some dosimetric materials for heterogeneous radiation sources. Radiat. Environ. Biophys..

[23] Şakar E., Özpolat Ö.F., Alım B., Sayyed M.I., Kurudirek M. (2020). hy-X/PSD: Development of a user friendly online software for calculation of parameters relevant to radiation shielding. Radiat. Phys. Chem..

[24] Hager I.Z., Rammah Y.S., Othman H.A., Ibrahim E.M., Hassan S.F., Sallam F. (2019). Nano-structured natural bentonite clay coated by polyvinyl alcohol polymer for gamma rays attenuation. J. Theor. Appl. Phys..

[25] Isfahani H.S., Abtahi S.M., Roshanzamir M.A., Shirani A., Hejazi S.M. (2019). Investigation on gamma-ray shielding and permeability of clay-steel slag mixture. Bull. Int. Assoc. Eng. Geol..

[26] Bashter I. (1997). Calculation of radiation attenuation coefficients for shielding concretes. Ann. Nucl. Energy.

[27] Olukotun S., Gbenu S., Ibitoye F., Oladejo O., Shittu H., Fasasi M., Balogun F. (2018). Investigation of gamma radiation shielding capability of two clay materials. Nucl. Eng. Technol..

[28] Sharaf J., Saleh H. (2015). Gamma-ray energy buildup factor calculations and shielding effects of some Jordanian building structures. Radiat. Phys. Chem..

